# Epigenetic Control of Plant Response to Heavy Metal Stress: A New View on Aluminum Tolerance

**DOI:** 10.3389/fpls.2020.602625

**Published:** 2020-12-16

**Authors:** Jenny Johana Gallo-Franco, Chrystian Camilo Sosa, Thaura Ghneim-Herrera, Mauricio Quimbaya

**Affiliations:** ^1^Departamento de Ciencias Naturales y Matemáticas, Pontificia Universidad Javeriana, Cali, Cali, Colombia; ^2^Grupo de Investigación en Evolución, Ecología y Conservación EECO, Programa de Biología, Facultad de Ciencias Básicas y Tecnologías, Universidad del Quindío, Armenia, Colombia; ^3^Departamento de Ciencias Biológicas, Universidad ICESI, Cali, Colombia

**Keywords:** abiotic stress, aluminum tolerance, epigenetic response, heavy metals, rice

## Abstract

High concentrations of heavy metal (HM) ions impact agronomic staple crop production in acid soils (pH ≤ 5) due to their cytotoxic, genotoxic, and mutagenic effects. Among cytotoxic ions, the trivalent aluminum cation (Al^3+^) formed by solubilization of aluminum (Al) into acid soils, is one of the most abundant and toxic elements under acidic conditions. In recent years, several studies have elucidated the different signal transduction pathways involved in HM responses, identifying complementary genetic mechanisms conferring tolerance to plants. Although epigenetics has become more relevant in abiotic stress studies, epigenetic mechanisms underlying plant responses to HM stress remain poorly understood. This review describes the main epigenetic mechanisms related to crop responses during stress conditions, specifically, the molecular evidence showing how epigenetics is at the core of plant adaptation responses to HM ions. We highlight the epigenetic mechanisms that induce Al tolerance. Likewise, we analyze the pivotal relationship between epigenetic and genetic factors associated with HM tolerance. Finally, using rice as a study case, we performed a general analysis over previously whole-genome bisulfite-seq published data. Specific genes related to Al tolerance, measured in contrasting tolerant and susceptible rice varieties, exhibited differences in DNA methylation frequency. The differential methylation patterns could be associated with epigenetic regulation of rice responses to Al stress, highlighting the major role of epigenetics over specific abiotic stress responses.

## Introduction

Plants deal with multiple challenges to adapt to different environmental conditions given their sessile lifestyle. Abiotic stresses such as drought, salinity, extreme temperatures, nutrient deficiency, and heavy metal stress, represent some of the most limiting factors for plant growth (Zhu, 2016).

Heavy metals (HMs) are elements with densities above 5g/cm^3^ that belong to the Earth’s crust natural components. High concentrations of heavy metals can generate cytotoxic, genotoxic, and mutagenic effects in living organisms. Under physiological conditions, HMs can be divided into two groups: (i). Essential elements that are necessary for plant growth being structural blocks in proteins with an enzymatic function, such as iron (Fe), manganese (Mn), zinc (Zn), magnesium (Mg), molybdenum (Mo), and copper (Cu), and (ii). Non-essential elements like Cadmium (Cd), chromium (Cr), lead (Pb), aluminum (Al), and selenium (Se). While essential elements are necessary for plants in small amounts, high concentrations of both types of elements can lead to inhibition of plant growth and development (Rascio and Navari-Izzo, 2011). Heavy metals have a strong impact on acid soils, caused by the excess of cationic species such as magnesium (Mg^2+^), calcium (Ca^2+^), phosphorus (P), sodium (Na^+^) and aluminum (Al^3+^) which in turn, affect plant physiological responses leading to crop yield losses for breeders and farmers (Samac and Tesfaye, 2003; Fryzova et al., 2017).

**FIGURE 1:**
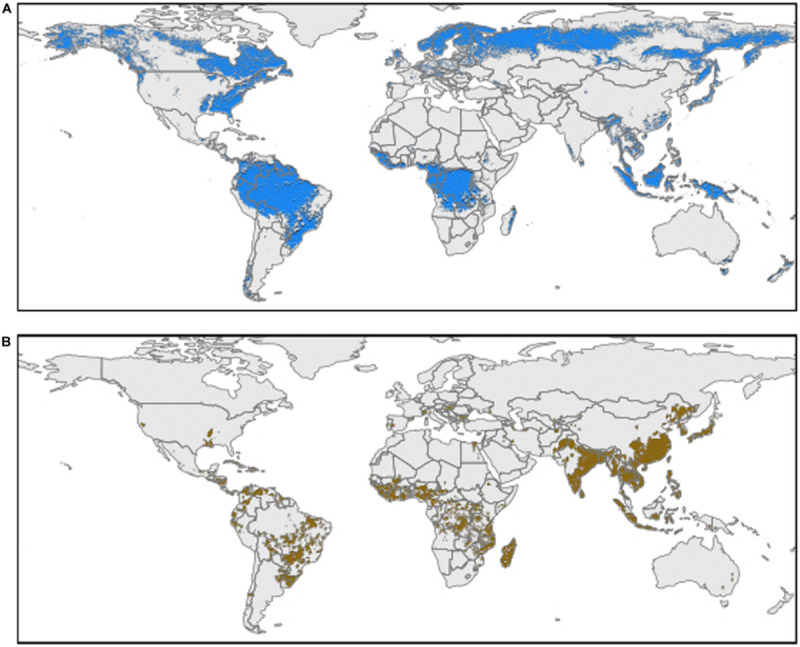
Worldwide distribution of acidic soils and rice crop areas (1 km^2^ resolution). **(A)** Areas with a weighted averaged soil pH (0-30 cm) less than or equal to 5.5 (acidic soils) using data extracted from Soilgrids (Hengl et al., 2017). **(B)** Worldwide rice crop area coverage (pixel probability > 0) (Jackson et al., 2019).

Acid soils represent nearly 30% of worldwide arable land, with 13% of staple crops cultivated in these areas. These types of soils classified as ultisols or oxisols are characterized by a pH lower than 5.5 (Figure 1A; Bojórquez-Quintal et al., 2017; Rahman et al., 2018). Al toxicity on acid soils has been reported as one of the major factors limiting crop production, and becoming worse due to current fertilization practices, pasture management, and climate change (Zheng, 2010; Kochian et al., 2015).

Staple food crops such as maize, wheat, sorghum, and rice have been extensively studied to increase their Al tolerance (Famoso et al., 2010). Among these crops, rice has been used as a model thanks to its high tolerance to Al toxicity (Famoso et al., 2010; Mustafa and Komatsu, 2016). Rice is a staple crop for over half of the world population with a cultivated area of 167.25 million hectares, and with an increment of 5.55 million hectares between 2010 and 2017 period (Food and Agriculture Organization of the United Nations, 2020; Figure 1B). Yet, there is still a need to increase 50% of rice production by 2050 to feed a growing population (Lin et al., 2019).

Important advances in elucidating the genetic mechanisms associated with HM tolerance and, especially, the molecular network involved in Al toxicity responses, have been reported in the last decade. Several studies on different crops have focused on genetic mapping to identify either quantitative trait loci (QTLs) or up/down-regulated genes associated with the response to Al stress (Famoso et al., 2011; Zhang et al., 2019). However, an increasing number of studies highlight the role of epigenetic mechanisms in the regulation of plant stress responses (Sudan et al., 2018; Chang et al., 2020). Therefore, the aim of this review is to explore and analyze the existing scientific literature on epigenetics as an important factor that regulates HM stress responses. Additionally, the direct relationship between epigenetic and genetic elements related to HM tolerance is revised, with a special focus on Al tolerance in rice.

## Genetic Mechanisms Underlying Heavy Metal Tolerance

**FIGURE 2:**
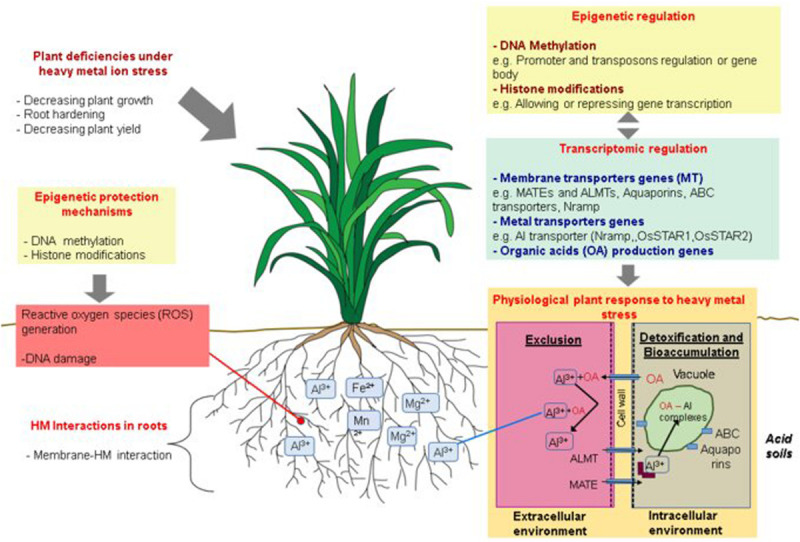
Schematic representation of physiological, genetic, transcriptional and epigenetic mechanisms involved in plant responses to heavy metals (HM) exposure. Plant exposure to HMs induces different physiological deficiencies that could be countered by two principal tolerance mechanism shown at the bottom right of the figure: an exclusion mechanism, where the plant secretes organic acids (OAs) out of the root, avoiding the entrance of HM ions or, a detoxification mechanism and sometimes bioaccumulation, wherein plants internalize HM ions through membrane transport proteins such as ALMT or MATE carriers, and subsequently, HMs can be chelated by organic acids (OA) or translocated into the vacuoles through ABC carriers or aquaporins. The regulation of HM responsive genes has been related to epigenetic mechanisms as DNA methylation and histone modifications which can repress or activate gene expression through promoter or gene body methylation as well as avoiding transposon movement (top right). Another important epigenetic mechanism involved in the HM stress response is the hypermethylation along the genome to protect DNA from possible damages caused by metal subproducts.

Plants have evolved different strategies to cope with HMs, diverging according to distinct factors as the plant species or the HMs exposure time and concentrations (Horst et al., 2010). These strategies fall into two general mechanisms: (i) An exclusion mechanism, where plants exudate organic compounds to the rhizosphere to chelate HM ions, transforming them into non-toxic compounds, and avoiding their chemical intake through root cells; and (ii) A detoxification mechanism, where plants allow the entrance of HM ions for internal detoxification and sequestration (Figure 2; Kochian et al., 2015).

Hyperaccumulator plants have been important models to understand the possible mechanism by which plants have adapted to high HM concentrations, and to elucidate the putative genetic elements that could be involved in these processes (Yang et al., 2005; Chaudhary et al., 2018; Fasani et al., 2018). One recurrent mechanism reported in these plants as an overall HM detoxification strategy is HM chelation by a ligand, either to keep HMs out of the roots or to target them to vacuoles. Diverse metal-binding ligands have been reported in plants. The peptide ligands phytochelatins (PCs) and metallothioneins (MTs) are different classes of cysteine-rich proteins that bind to HMs and have been reported as the most important genes in HM detoxification (Chaudhary et al., 2015). Complexes of PC-HM lower the binding capacity of HMs to the cell walls while MTs control the ROS accumulation and HM sequestration. For more information see Chaudhary et al. (2018) for a review of different PC and MT genes expressed in various plants and tissues under different HM stresses. Another mechanism involved in HM tolerance is the HM transport into the cell, and later, into the vacuole. Various genes have been reported to be involved in HM transport including heavy metal ATPases and the natural resistance-associated macrophage protein (Nramp) (Yang et al., 2005; Chaudhary et al., 2015).

Several studies have reported that tolerance or hyperaccumulation of HMs in plants is related to gene transcription modulation of metal chelators or transporters that favor exclusion or detoxification of the HMs (Arbelaez et al., 2017; Gulli et al., 2018; Zhang et al., 2019). These genes are potentially regulated by a reversible epigenetic mechanism, especially on hyperaccumulator plants which can live in soils with or without high HM concentrations. In this sense, epigenetic mechanisms represent an option to modify gene expression patterns enabling a rapid adaptation to environmental stressors (Mirouze and Paszkowski, 2011; Ou et al., 2012). Table 1 shows the main genetic players in plant responses to Al, including genes involved in the exclusion or sequestration of Al^3+^ ions.

**TABLE 1:** Summary of main exclusion and tolerance mechanisms reported in plants.

**Species**	**Genes**	**Mechanism**	**Specific mechanism**	**Function**	**References**
*P. vulgaris, T. aestivum, S. bicolor, H. vulgare, Zea mays*, snapbean, oat, rye, *Glicine ma*x, *Colocasia esculenta, Triticale* sp., *Helianthus annuus*	ALMT, MATE, OSALMT4	Exclusion	Organic acid exudation	Chelate Al3+ (release of malate, citrate, or oxalate) located in the root apex	Kochian et al., 2004, 2015; Liu et al., 2018
*Zea mays*, *Cinnamomum camphora, Eucalyptus camaldulensis*		Exclusion	Phenolic compounds exudation	Release of other organic compounds (e.g., catechol, catechin, and quercetin), oenothein B, proanthocyanidin in roots	Kochian et al., 2015
*Cucurbita pepo*, wheat, tea	ATPases	Tolerance (Al detoxification)	Changes in the Rhizosphere pH	pH rhizosphere changes to induce to Al detoxification mechanisms	Bojórquez-Quintal et al., 2017
*Oryza sativa, Solanum tuberosum, Arabidopsis thaliana, petunia inflata*	XTH, XET, XTH31,pectin methylesterases,OsFRDL4, STAR1, STAR2, ABC transporters, HMG2, HMG3, WAK1	Tolerance (Al detoxification)	Cell wall modification	Changes in the structural properties of cell wall such as reduction of wall plasticity/elasticity, carbohydrates, methylated pectins, and reduced pectin methylesterases; increased sterols biosynthesis; negativity of apoplast to enhance Al transport	Schmohl et al., 2000; Horst et al., 2010; Kochian et al., 2015; Morkunas et al., 2018; Wagatsuma et al., 2018
*Arabidopsis thaliana, Oryza sativa*,	Nramp, OsNrat1, OsALS1, aquaporine family, ABC,ALMT,OsCDT3	Tolerance (Al detoxification)	Al transportation	Arrest Al from cell wall to root cell vacuole	Kochian et al., 2015; Arbelaez et al., 2017
*Brassica napus, Nicotiana tabacum*, wheat, *Arabidopsis thaliana, Zea mays*	ALMT,MATE,SbMATE, TaALMT1,OsFRDL4	Tolerance (Al detoxification)	ALMT/MATE proteins Al transportation	Passive efflux of malate; carriers that mediate citrate efflux coupled to H + influx	Liu et al., 2014; Kochian et al., 2015
*Oryza sativa, Arabidopsis thaliana, Andropogon virginicus*	Nramp,OsALS1, Nrat1	Tolerance (Al detoxification)	Nramp proteins Al transportation	Specific transporter for aluminum ions (no divalent cations) transport from cell wall to vacuoles	Yokosho et al., 2011; Ezaki et al., 2013; Kochian et al., 2015
*Oryza sativa, Arabidopsis thalian*a	OsSTAR1, OsSTAR2, AtALS3, OsALS1, AtALS1	Tolerance (Al detoxification)	ABC proteins Al transport	ATP-driven pumps (ABC transporters);	Huang et al., 2009; Delhaize et al., 2012; Kochian et al., 2015
*Oryza sativa, Arabidopsis thaliana, Hydrangea macrophylla*	Aquaporins such as HmVALT, HmPALT1	Tolerance (Al detoxification)	Aquaporins transportation	Transport and store in shots	Negishi et al., 2012; Kochian et al., 2015

*The relationship among genes, mechanisms and molecular functions of the reported genes is shown.*

One of the main strategies reported for Al exclusion is mediated by organic acid (OA) efflux from the root apex (Yang et al., 2013; Poschenrieder et al., 2019), a ubiquitous mechanism in all plant cells that reduces Al damage by forming stable compounds with Al^3+^ ions in the rhizosphere (Bojórquez-Quintal et al., 2017). The first genes linked to Al tolerance were malate and citrate organic acid transporters in wheat (*Triticum aestivum*), sorghum (*Sorghum bicolor*), and barley (*Hordeum vulgare*) (Sasaki et al., 2004; Furukawa et al., 2007; Magalhaes et al., 2007). Subsequently, it was found that members of two transporters families, the Al-activated malate transporter (ALMT) and the OA/H + transport channel (Multi-antimicrobial extrusion protein - MATE), are responsible for the exudation of malate and citrate, respectively, from root cells to the rhizosphere in response to Al (Kochian et al., 2015). However, other transporters like ABC carriers and aquaporins are also required for OA transport (Liu et al., 2014).

## Rice as a Genetic Model to Study Aluminum Tolerance in Plants

Rice is a model species to study Al tolerance being one of the plants with highest tolerance to this element (Famoso et al., 2010, 2011). Rice has a complex response against Al stress, involving a wide range of strategies and a diversity of genes (Magalhaes et al., 2004). These genes are potentially involved in the exclusion of Al^3+^ ions through OA efflux; for instance, the MATE transporters OsFRDL2 and OsFRDL4, has shown a role in OA transport (Famoso et al., 2010; Delhaize et al., 2012; Yokosho et al., 2016). Other rice Al responses include the modification of the cell wall properties (Kochian et al., 2015; Che et al., 2016), and Al^3^*^+^* ions uptake and subsequent sequestration/translocation into the vacuole by different Al transporters like bacterial-type ABC and Nramp Al transporters (Huang et al., 2009; Xia et al., 2010; Li et al., 2014). Other genetic elements associated with Al tolerance include genes encoding transcription factors as ART1, ASR1 and ASR5 (Yamaji et al., 2009; Arenhart et al., 2016; Che et al., 2016). The upregulation of specific genes as OsMGT1, a magnesium transporter, is also linked to high Al tolerance (Chen et al., 2012). More recently, Zhang et al. (2019) reported 69 potential candidate genes related to Al tolerance, identified in a collection of 150 rice landraces using a combined GWAS-transcriptomic approach. Complementarily, several QTLs associated with Al tolerance have been identified in rice using different inter and intra-specific mapping populations (Wu et al., 2000; Ma et al., 2002; Nguyen et al., 2003; Xue et al., 2006, Xue et al., 2007; Famoso et al., 2011; Zhang et al., 2019). Famoso et al. (2011) reported 48 QTLs located on chromosomes 1, 3, 9, and 12. The QTLs were generated based on mapping populations exposed to Al stress, using relative root growth as the experimental phenotypic readout. The major QTL was found on chromosome 12, explaining 19% of the phenotypic response. Findings reported in above mentioned studies support the hypothesis that Al tolerance in rice involves multiple genes, genomic regions and mechanisms.

The previous evidence relates both, genic elements and specific genic mechanisms with the phenotypic response to cope with HMs stresses. Besides the genetic control that exists to regulate these responses, additional regulation layers might exist, being epigenetics a controlling mechanism of paramount importance in order to adapt to abiotic stresses, and specifically, to HMs restrictive conditions. In the following sections we will revise the current evidence that associates epigenetics with HMs stress responses. Giving its agronomic relevance, special attention is put on rice epigenetics as integrated strategies to cope with HMs and aluminum stresses.

## Epigenetic Mechanisms in Plants

Epigenetics refers to the study of heritable and stable changes in gene expression without DNA sequence modifications (Wu and Morris, 2001). Three epigenetic mechanisms have been described in gene expression regulation: (i) DNA methylation (modifications at genomic level), (ii) histone modifications (chromatin modifications) and (iii) Small RNA modifications (RNA directed DNA Methylation-RdDM pathway) (Sudan et al., 2018; Chang et al., 2020). Currently, DNA methylation is the most documented epigenetic modification, and it is recognized as a relatively stable, and inheriting transgenerational mark involved in a set of biological processes such as the activity of transposable elements, genomic imprinting, alternative splicing, and regulation of temporal and spatial gene expression (Zhang et al., 2006; Ou et al., 2012). Mammals and plants differ in their DNA methylation patterns. In plants, DNA methylation is more widespread and complex, and occurs mainly in cytosine residues in the CG, CHG, and CHH sequence context (H can be A, C, or T), while in mammals it occurs only in a CG context (Bender, 2004; He et al., 2010). Studies on general DNA methylation profiles conducted on the model crop, *Oryza sativa* L. (cultivated rice), have shown that transposable elements and repetitive sequences are the most heavily methylated DNA regions in the rice genome (He et al., 2010; Yan et al., 2010; Li et al., 2012). Overall, gene methylation occurs mainly in the CG context, while transposon methylation occurs in all three described contexts (He et al., 2010; Yan et al., 2010; Li et al., 2012).

The methylome in plants is mainly monitored and maintained during DNA replication and cell division by DNA methyltransferases. There are three major classes of DNA methyltransferases: DNA methyltransferases (METs), which are the main CG methylases in charge of CG methylation; the plant specific enzymes chromomethyltransferases (CMTs), that are known to establish CHH and CHG methylation; and the domain rearranged methyltransferases (DRMs), that are involved in the maintenance of non-CG methylation and *de novo* methylation in all three contexts: CG, CHG and CHH (Lanciano and Mirouze, 2017). In contrast, DNA demethylation is performed by DNA glycosylases such as ROS1 (Repressor Of Silencing 1) and the DME (Demeter) enzyme (Lanciano and Mirouze, 2017).

## Epigenetic Regulation of Plant Stress Response

Abiotic stresses can generate a diverse range of phenotypes in plants, which are a consequence of complex molecular, biochemical, and physiological changes. Plants responses and adaptation to these stress conditions vary in different ways and at various levels, including short term physiological responses such as metabolic and gene expression changes, and long-term responses such as genetic and epigenetic genome modifications (Turner, 2009). The mechanisms of signal transduction, as well as the genetic variability underlying plants responses to stress, have been widely studied and, in many cases, successfully exploited by plant breeders to improve resistance to abiotic stress through traditional breeding or marker-assisted selection (Kantar et al., 2015; Zhu, 2016). Recently, epigenetic marks have gained attention as important factors of abiotic stress-related gene control (Kumar, 2018). For example, a stress signal can promote DNA methylation changes in the promoter regions of stress-responsive genes, thus modifying their expression pattern, generating histone conformational changes, and promoting transcriptional repression by preventing transcription factors binding to their target sites (Boyko et al., 2010; Ou et al., 2012; Ueda and Seki, 2020). Since methylation affects how genes are transcribed, it is hypothesized that DNA methylation is involved in the long-term transgenerational maintenance of epigenetic changes.

DNA methylation states can be complemented by additional mechanisms such as histone modifications (Mirouze and Paszkowski, 2011). Although considered a more dynamic and transitory mechanism, because the majority of changes that occur under stress conditions revert to their initial state quickly, histone modifications could play a role in the inheritance of certain stress-tolerant phenotypes (Pecinka and Scheid, 2012). For example, Kim et al. (2012) showed that H3K4me3 and H3K9ac histone modifications were abundant in several drought-associated genes in *Arabidopsis thaliana* plants subjected to water-deficit regimes. When plants were irrigated, the H3K9ac modifications were rapidly eliminated, while H3K4me3 ones remained, indicating that the latter modification can be stably inherited through generations.

Histone modification effects on gene regulation have also been reported for other stress conditions. Sokol et al. (2007) reported transient H3Ser-10 phosphorylation, H3 phosphoacetylation, and histone H4 acetylation under salinity and cold-stress related to the expression of stress-specific genes. Likewise, the trimethylation of H3K4 and acetylation of H3K9 in *A. thaliana* was generated by exposure to drought, ABA, and salt stress, causing stress-responsive genes expression (Kim et al., 2008).

Stress-induced epigenetic changes, especially DNA methylation, occur regularly in all plant species, reinforcing the importance of this mechanism for regulating plant responses to environmental changes; most of these changes are heritable and play an important role in plant adaptation (Feng et al., 2010). Genomic sequences whose changes in their methylation status are maintained over generations, without altering the acquired methylated pattern, are known as epialleles (Kalisz and Purugganan, 2004). There is evidence that epialleles can occur over stress-related genes, however, they can also be present in genetic regions that are not directly related with the specific stress response, generating random changes across the genome. Moreover, both types of variations could be affected by natural selection according to the phenotypic effects they may cause (Verhoeven et al., 2010).

Transposons can also play a role in suppressing gene expression. This can occur due to the methylation state of a transposon located in or near a gene, which can directly affect the regulation of that gene through a methylation spread mechanism. Thus, transposon silencing through epigenetic marks contributes to the establishment of epigenetic variations affecting gene modulation in plants (Saze and Kakutani, 2007; Galindo-González et al., 2018).

Although the heritability of stress-induced methylation in plants remains poorly understood, some studies show that most of the induced variations are faithfully inherited to the offspring. For instance, Boyko et al. (2010) showed that *A. thaliana* plants exposed to salinity, cold, heat, and flooding, showed an overall increase in DNA methylation, associated with a higher stress tolerance in the progeny. In addition, Herman and Sultan (2016) reported that in *Polygonum persicaria*, DNA methylation is involved in increasing offspring drought tolerance when parental plants are subjected to this stress. Some studies have even found epialleles with direct effects on economically important traits; for instance, heritable methylation changes induced in rice due to nitrogen deficiency (Kou et al., 2011), heavy metal toxicity (Ou et al., 2012), and drought (Zheng et al., 2017) have been described. This last study showed the conservation of several non-random methylation changes generated under drought conditions (>40%) through several generations. Zheng et al. (2017) also found that these epigenetic changes are related to stress-responsive genes and they seemed to influence rice long-term adaptation to drought conditions. Thus, these studies support the potential role of epigenetic variation, and its inheritance across generations, as a relevant evolutionary process in crops. Similarly, they show that in rice, the mechanisms of epigenetic regulation of stress responses may be related to the type of stressor.

## Epigenetic Mechanisms Involved in Heavy Metal Toxicity

A recent recurring question is whether there is a general pattern of DNA methylation related to HMs exposure in plants. Evidence from previous studies suggests that DNA methylation might play a role in the regulation of plant responses to HMs through at least two mechanisms (Aina et al., 2004; Choi and Sano, 2007; Greco et al., 2012; Kumar et al., 2012; Arif et al., 2016). The first mechanism is related to a protective effect of methylation against HM-induced DNA damage through single-strand breaks or multi-copy transposition (Figure 2; Bender, 1998). For example, Aina et al. (2004) compared methylation levels between clover (*Trifolium repens L*.), which is sensitive to Cr, Ni, and Cd, and hemp (*Cannabis sativa* L.), which is partially tolerant to these HMs. The study found that in the absence of HM stress, the level of methylation of hemp roots was significantly higher than in clover. Similarly, Gulli et al. (2018) found that *Noccaea caerulescens* plants (a Ni hyperaccumulator species) grown under high Ni concentrations were significantly hypermethylated at the genome level in comparison to *A. thaliana* Ni susceptible plants exposed to high Ni concentrations. These authors also showed that MET1, DRM2, and HDA8 genes, which are involved in DNA methylation and histone modification, were differentially expressed between *N. caerulescens* and *A. thaliana.* Hypermethylation has also been reported to act as a defense mechanism to counteract radiation genotoxic effect as shown by Kovalchuk et al. (2003); Volkova et al. (2018) who reported that pine trees plants (*Pinus silvestris*) adapted to survive high ionizing radiation, exhibited significantly hypermethylated loci compared to less adapted plants.

A second type of epigenetic response to HM stresses involves gene expression control (Figure 2). This regulation is not limited to the promoter region of genes but includes their coding regions (Choi and Sano, 2007). DNA methylation on gene promoters usually represses genetic transcription but, in some cases, it can also promote it (Zhang et al., 2006). In the meantime, exon/intron methylation occurs mainly on CG context and its function remains unclear. Gene body methylation has been related to transcriptional upregulation and has been suggested to protect genes from aberrant transcription caused by cryptic promoters (Zhang et al., 2006; Feng et al., 2016). The local acetylation of histones located near the promoter region of genes can induce transcriptional activation (Finnegan, 2001). Although there are no reports of specific histone modifications related to HM stresses in plants, some studies in animals have revealed a direct relation between HM exposition and histone modifications (Cheng et al., 2012).

Gene expression changes generated by HM exposure in rice have been described extensively in the literature and linked to variations in DNA methylation levels. For instance, Oono et al. (2016) showed a positive correlation between Cd dose-response in plants and the expression of genes coding for metal ion transporters where DNA methylation marks were detected. Similarly, using whole-genome bisulfite sequencing (WGBS), Feng et al. (2016) evaluated DNA methylation changes induced by specific Cd stress in rice plants (*Oryza sativa* ssp japonica cv. Nipponbare). The authors found specific differentially methylated regions after Cd treatment, with patterns of methylation closely associated with transcriptional differences of stress response genes involved in metal transport, metabolic processes and transcriptional regulation. Likewise, some studies have shown the heritability and stability of HM stress-induced methylation changes (Rahavi et al., 2011; Ou et al., 2012). For instance, in *A. thaliana*, improved tolerance to HMs has been observed in the progeny under the same stress experienced by parental plants (Ou et al., 2012). More recently, Cong et al. (2019) showed that specific methylation changes induced by HM stress, specifically methylation changes at the Tos17 retrotransposon, displayed transgenerational inheritance through three generations. Therefore, the evidence suggests that epigenetic mechanisms contribute to HM stress adaptation through successive plant generations.

**Box 1. Methods to study DNA methylation.**

***metAFLP (Amplified fragment length polymorphism)*** – *metAFLP* is a variation of the AFLP method. Nowadays it is poorly implemented given the emergence of genomic-scale methods. It is a cost-effective methodology that was used to elucidate methylation patterns in plants. The technique is able to detect global methylation marks throughout the studied genome. It is based on isoschizomers implementation to cut the DNA inside specific sites that display differential sensitivity to DNA methylation. A fragment comparison analysis reveals specific methylation polymorphisms. A major limitation is that it can only assess a small percentage of a global DNA methylation scenario. An important advantage is that these methods can be used for any species, even with limited or no information about their DNA sequence composition (Bednarek et al., 2007). ***MSAP (Methyl Sensitive Amplified Polymorphism)*** – This technique is a modification of the *metAFLP* technique described above. The protocol uses the *Eco*RI restriction enzyme in combination with the methylation-sensitive enzymes *Hpa*II and *Msp*I. These last isoschizomers recognize and cleave the same tetranucleotide sequence 5′-CCGG, but differ in the sensitivity to cytosine methylation. The method can differentiate among methylated, hemimethylated, or non-methylated sites. This technique was broadly implemented because of its cost-effective advantages, but one of its principal limitations is that it cannot specify the region or gene influenced by methylation (Bednarek et al., 2017).

***CRED-RA (Coupled restriction enzyme digestion and random amplification)*** – Similar technique as the ones previously described. It is based on the use of restriction enzymes, specifically the isoschizomers *Hpa*II and *Msp*I implemented as Random Amplified Polymorphic DNA (RAPD) (Erturk et al., 2015).

***HPLC (high-performance liquid chromatography)*** – There are several variants for this methodology but in general it involves the enzymatic hydrolysis of DNA to its deoxyribonucleotide components and subsequent separation and quantification of the nucleotides by high-performance liquid chromatography. The system gives highly reproducible results and, under suitable conditions, it is capable of measuring 5-methylcytosine levels even at low DNA concentrations. This method is implemented by comparing control samples versus treatments to evaluate genome-wide methylated cytosines. A major drawback is that the method is incapable of determining the sequence context of the methylated cytosine (Ramsahoye, 2002).

***WGBS (Whole-genome bisulfite sequencing)*** – It is considered as the “gold standard” method in DNA methylation studies. This technique is based on whole-genome sequencing protocols, after bisulfite conversion of DNA. The bisulfite DNA treatment mediates the deamination of non-methylated cytosines into uracil, and these converted residues will be read as thymine, after subsequent high throughput sequence analysis. The main limitations are cost and bioinformatic analysis of NGS data, which can be overcome with reduced representation bisulfite sequencing (RRBS), where only a genome fraction is sequenced (Kurdyukov and Bullock, 2016).

## Epigenetic Mechanism Involved in Aluminum Toxicity

Al exposure can trigger DNA damage and cell death through a strong binding of Al ions to pectins and other structural components of the cell wall (Murali Achary and Panda, 2010). Although there are currently few studies that have explored the relationship between epigenetic regulation and aluminum tolerance (Table 2), current evidence suggests that Al tolerance might be conferred through DNA methylation as specific methylation changes frequently occur after Al exposure. For example, Bednarek et al. (2017) subjected five Al-tolerant and five non-tolerant triticale lines to Al exposure. Using methylation-sensitive amplification polymorphisms (MSAP) (Box 1), the study showed that Al exposition in both Al-tolerant and non-tolerant plants induced demethylation. These findings are consistent with other reports that describe the effects of HMs on methylation patterns (Aina et al., 2004; Filek et al., 2008; Ou et al., 2012; Feng et al., 2016). However, the opposite pattern has also been reported; for example, by using coupled restriction enzyme digestion and random amplification (CRED-RA) in corn (*Zea mays* cv. RX9292), Taspinar et al. (2018) established that exposure to Al induced mobilization of long terminal repeat retrotransposons (LTR) and triggered DNA hypermethylation as a protective response to the stress condition. Complementarily, Agnieszka (2018) compared liquid chromatography (RP-HPLC), MSAP analysis and methylation amplified fragment length polymorphisms (metAFLP) (Box 1) to detect DNA methylation levels of triticale lines showing contrasting tolerance to Al treatments. After Al exposure, a reduction in DNA methylation across non-tolerant lines was identified with the RP-HPLC technique, in contrast, increased methylation was seen in tolerant plants; this outcome was independent of the Al dose. When MSAP was used, increased demethylation was found in the roots of both, non-tolerant and tolerant lines, with no differences between them. Finally, metAFLP results demonstrated no differences in DNA methylation under stress conditions, suggesting that only a portion of the genome responds to Al stress.

**TABLE 2:** Summary of epigenetic studies related to aluminum stress responses in plants.

**Plant**	**Variety**	**Epigenetic modification**	**Method**	**References**
*Nicotiana tabaccum*	*Xan-thi* nc	DNA methylation	HPLC, direct bisulfite sequencing	Choi and Sano, 2007
*Sorghum bicolor*	*inbred lines, YN336 and YN267*	DNA methylation	MSAP	Kimatu et al., 2011
*Zea Mays*	*Kenyan tropical maize (KTM)*	DNA methylation	MSAP	Kimatu et al., 2013
*Arabidopsis thaliana*	*Col-0 ecotype*	DNA methylation, histone modifications	Chromatin Immuno-precipitation (ChIP), direct bisulfite sequencing.	Ezaki et al., 2016
*Triticale* inbred lines		DNA methylation	MSAP	Bednarek et al., 2017
*Zea mays*	*cultivar RX9292*	DNA methylation	CRED–RA	Taspinar et al., 2018
*Triticale* inbred lines		DNA methylation	metAFLP, MSAP, HPLC	Agnieszka, 2018
*Triticum aestivum*	*Haymana 79, Kılçıksız, and Bezostaja 1*	DNA methylation	CRED-iPBS	Pour et al., 2019

Pour et al. (2019) used CRED_RA in three wheat cultivars (cv. Haymana79, Kılçıksız, and Bezostaja1) to evaluate genetic and epigenetic variations to different Al conditions (7.5 and 30mM). DNA hypermethylation was observed in wheat plants at higher Al concentration (30 mM) and hypomethylation at lower Al concentration (7.5 mM). These results suggest a gradual effect of Al on methylation, with concomitant cellular damages associated with increased Al toxicity. A methylation increase along the genome was concluded to confer a protective response in the affected plants. Thus, the existing evidence points to a complex influence of DNA methylation on the response to Al-induced stress in a species-dependent manner.

Methylation changes caused by Al exposure can be targeted to specific genomic locations. Choi and Sano (2007) showed a direct effect of Al over methylation changes in stress response genes in wild tobacco plants (*Nicotiana tabaccum* cv Xan-thi nc). The study showed that Al stress promotes demethylation in the coding region of the glycerophosphodiesterase-like protein gene (NtGPDL) resulting in enhanced expression. NtGPDL belongs to the glycosylphosphatidylinositol-anchored protein (GAP) family linked to the extracellular matrix. Although the function of this gene is unclear, it seems to be involved in stress responses, including Al stress in tobacco (Borner et al., 2003). Similarly, in transformed S-adenosylmethionine (SAM) *Arabidopsis* plants. The inserted gene derived from the Al-tolerant plant, *Andropogon virginicus* (AvSAMS1), conferred enhanced Al tolerance to *A. thaliana*. This enzyme represents the main methyl group donor in plants and appears to play an important role in the epigenetic stress response. Overexpression of the AvSAMS1 resulted in changes both in DNA and histone H3 methylation after plant exposure to Al. More interestingly, there were differences in the demethylation and methylation patterns at different positions in the promoter and coding regions of this gene (Ezaki et al., 2016).

Transposable elements play a role in Al stress responses. Kashino-Fujii and colleagues analyzed Al-tolerant accessions of barley derived from a multi-retrotransposon-like (MRL) insertion, located upstream of the coding region of the HvAACT1 gene. This gene is responsible for citrate efflux in roots, a mechanism involved in Al detoxification. The MRL insertion acted as a promoter and significantly enhanced HvAACT1 expression in Al-tolerant plants. This study showed that both the MRL insertion and gene expression, are due to demethylation processes, and are necessary for Al tolerance in barley. Additionally, transposon insertions close to genes have been proposed as a source of epialleles, and as a mechanism affecting the transcriptional regulation of specific genes (Slotkin and Martienssen, 2007; Kashino-Fujii et al., 2018). Moreover, methylation would have a role in controlling genes associated with Al tolerance in plants.

## DNA Methylation as a Regulatory Factor in Plant Responses to Aluminum Stress: Rice as a Study Case

Epigenetics has the potential to explain mechanistically, at least part of the molecular responses to different abiotic stresses, including HM toxicity (Figure 2). Although there are no studies related to the epigenetic regulation of Al tolerance in rice, we hypothesize that epigenetic mechanisms, like DNA methylation, could play an important role as a regulatory factor in this response. Potentially, several of the genes mentioned in this review might be regulated through differential patterns of DNA methylation. To test this assumption, we performed a brief analysis to quantify the methylation status of specific Al responsive genes in three different rice varieties (IR64, Nipponbare, and Pokkali) with contrasting responses to Al exposure.

**FIGURE 3:**
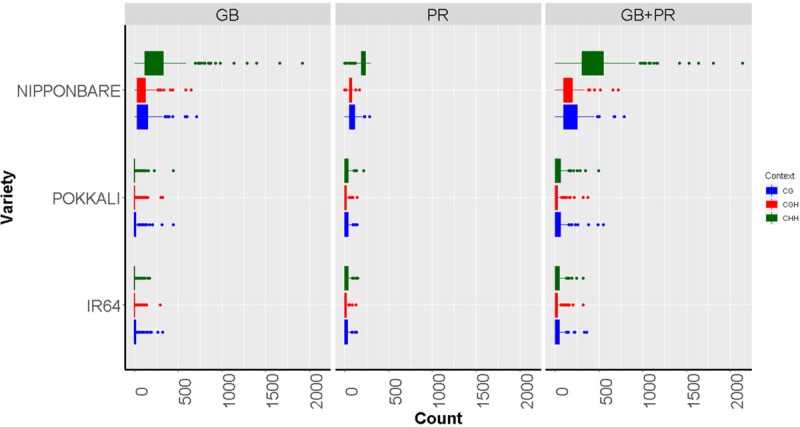
Boxplots showing methylated cytosine frequency in three sequence contexts: CG (blue), CHG (red), and CHH (green) among three different rice varieties with contrast responses to aluminum exposure: Nipponbare (Tolerant), Pokkali, and IR64 (Susceptible). The results are discriminated according to the location of the epigenetic mark, either inside the gene body region (GB), the promoter (PR), or both the promoter and inside the gene body region of analyzed genes (PR + GB).

For this evaluation, we analyzed publicly available data from Stroud et al. (2013) obtained from the Nipponbare cultivar (highly tolerant to Al toxicity) and from Garg et al. (2015) for IR64 and Pokkali varieties (susceptible to Al toxicity). To explore the possible role of methylated cytosines over gene expression, in a set of 250 genes associated with Al tolerance in rice (Arenhart et al., 2014; Arbelaez et al., 2017), we calculated the number of methylated cytosines considering the different methylation contexts (counting was performed 1000 bps before and after the transcription initiation site). According to the reported experimental data, these 250 genes showed significant changes in expression after Al exposure (upregulated genes Log2FC ≥ 1, downregulated genes Log2FC ≤ −1) (Supplementary Table 1). Additionally, to increase the probability that the effects over gene expression were caused by an epigenetic regulation solely, we filtered out from this list, those genes with differences in copy number or with SNP variations in the coding region, retaining for the analysis only single-copy genes identified from the rice genes paralogous list generated by Lin et al. (2008) and without SNPs variants identified from the database Rice SNP-Seek database (Mansueto et al., 2017^1^). As a result, a group of 72 genes was kept, representing 10% of genes with the highest counts for methylated cytosines (Supplementary Table 2). After filtering by gene duplication and SNPs variants, we retained 26 candidate genes (Supplementary Figure 1 and Supplementary Table 3). Among the three analyzed varieties, taking into account the different methylation contexts, and the localization of the methylated cytosines, Nipponbare exhibited more methylated sites than the other two varieties (*p* ≤ 0.01 in an FDR analysis), while IR64 and Pokkali did not show differences in methylation (Figure 3). These results are interesting since Nipponbare has been extensively reported as a cultivar highly tolerant to Al (Famoso et al., 2010).

At the top of the list, representing highly methylated genes (Table 3), we found some genes previously reported as important players in rice Al tolerance. For example, the Calmodulin binding protein (Loc_Os09g13890) is a calcium ion-binding molecule that regulates different cellular processes, and recently, the association of the Calmodulin signal transduction pathway to Al stress has been reported (Zhang et al., 2016). This study showed that transgenic *Saccharomyces cerevisiae* strains transformed with the Calmodulin gene were more tolerant to Al toxicity, suggesting that the gene is a good candidate for improving Al tolerance in plants through transgenic approaches. Similarly, our analyses also showed the proteins STAR1 (Loc_Os06g48060) and ART1 (Loc_Os12g07280) as relevant in Al-related methylation. STAR1 encodes a nucleotide-binding domain that associates with STAR2, which encodes a transmembrane domain, to form a bacterial-type ABC transporter required for Al detoxification in roots (Table 1; Huang et al., 2009). On the other hand, the ART1 zinc finger protein is a transcription factor that regulates around 31 genes, probably involved in Al detoxification at different cellular levels, including STAR1 and STAR2 genes (Yamaji et al., 2009). Our results suggest that the methylation status of reported Al response genes, could play a role in Nipponbare’s Al tolerance.

**TABLE 3:** Top 10 of genes with the highest methylated cytosines counts for three *O. sativa* varieties with different Aluminum tolerance levels.

**Gene (MSU id)**	**Annotation**	**IR64**	**Nipponbare**	**Pokkali**
Loc_Os12g32850	Cytochrome P450 71E1, putative	202	949	273
Loc_Os09g13890	Calmodulin binding protein, putative, expressed	202	1075	159
Loc_Os12g42860	Cysteine dioxygenase	161	937	219
Loc_Os03g11950	CRAL/TRIO domain containing protein, expressed	137	1059	156
Loc_Os06g48060	Protein STAR1	130	1155	175
Loc_Os05g51470	2-aminoethanethiol dioxygenase, putative, expressed	115	1053	143
Loc_Os12g07280	Zinc finger protein ART1	109	1024	99
Loc_Os12g06660	Actin-7, putative, expressed	99	990	121
Loc_Os04g33640	Glycosyl hydrolases family 17, putative, expressed	83	1357	94
Loc_Os09g37510	DUF292 domain containing protein, expressed	69	941	82

*Annotations were performed using the uniprot database.*

## Aluminum Beneficial Effects for Plants

Although Al has been mainly studied for its toxic effects on plants, it can also generate benefits by inhibiting other toxic minerals, increasing defense against pathogens and by stimulating the absorption of specific nutrients as Mg, Ca, K, and P (Bojórquez-Quintal et al., 2017). Likewise, several reports show that Al can stimulate growth of both, plants adapted to acid soils (Gulli et al., 2018; Muhammad et al., 2019), and growth of commercially important crops as rice (Famoso et al., 2011) and corn (Wang et al., 2015). In plants like tea the presence of Al in soil stimulates root growth whereas its absence results in stunned plants (Fung et al., 2008). Both beneficial and negative effects are related to Al availability (Bojórquez-Quintal et al., 2017).

Some beneficial effects generated by Al are consequences of Al^3+^ cellular interactions. For example, organic acids that are exudated as a response to Al exposure, promote root growth and can increase the availability and uptake of P when it is present at limiting conditions (Muhammad et al., 2019). Currently, there are no reports of epigenetic mechanisms directly related to positive responses to Al toxic conditions, but it is possible to hypothesize that the epigenetic regulation of genes associated with the biosynthesis of organic acids, can indirectly and positively influence tolerant phenotypes in certain plants. Likewise, there are many other genes involved in metabolic processes as antioxidant enzymes, for which changes in their expression can be epigenetically regulated (Bojórquez-Quintal et al., 2017).

## Conclusion and Perspectives

Current knowledge of HM and Al tolerance in plants has been extensively documented with a direct focus on the physiological, and biochemical effects of these molecules, and their negative impacts on crop production. In rice, there is abundant information about genes and QTLs involved in Al tolerance in comparison with other staple cultivars such as barley or even the model plant *A. thaliana*. Nevertheless, recently, epigenetic mechanisms have emerged as important factors in the response of plants to HM stresses. Two main epigenetic strategies are relevant: (i) epigenetic marks are used as a mechanism to protect plants from possible DNA damage caused by metal ions through random DNA methylation along the genome, and (ii) epigenetic changes are used for the regulation of transposon and stress-responsive genes (Figure 2).

The studies carried out so far are evidence of putative epigenetic changes caused by HM exposure. However, it is necessary to evaluate the patterns of DNA methylation, as well as histone modifications occurring in precise genome regions to understand the possible epigenetic mechanisms underlying the regulation of the complex gene networks of Al tolerance responses. Likewise, there is a need for development of bioinformatics pipelines for epigenetic analyses. Future studies will be mandatory to evaluate the stability of the reported epigenetic changes through generations, given that epialleles can become permanent marks affecting genotypes and phenotypic responses. Finally, we report an overall greater abundance of methylated cytosines in an Al-tolerant rice variety, showing a contrasting methylation pattern related to differentially expressed Al responsive genes. This supports the hypothesis of DNA methylation as a fundamental key factor in the rice response to Al exposure.

## Author Contributions

JG-F and CS performed the methylation analysis on Nipponbare, IR64, and Pokkali, and wrote and checked the manuscript. TG-H and MQ designed, edited, and checked the manuscript. All authors contributed to the article and approved the submitted version.

## Conflict of Interest

The authors declare that the research was conducted in the absence of any commercial or financial relationships that could be construed as a potential conflict of interest.

## Footnotes

1 https://snp-seek.irri.org/
