# Supplementary material for: Epigenetic Control of Plant Response to Heavy Metal Stress: A New View on Aluminum Tolerance
Source: Front Plant Sci. 2020 Dec 16;11:602625. doi: 10.3389/fpls.2020.602625 (PMC7772216; doi:10.3389/fpls.2020.602625)
Supplement: Supplementary file 1 [file Table_1.docx]

***Supplementary material***

***
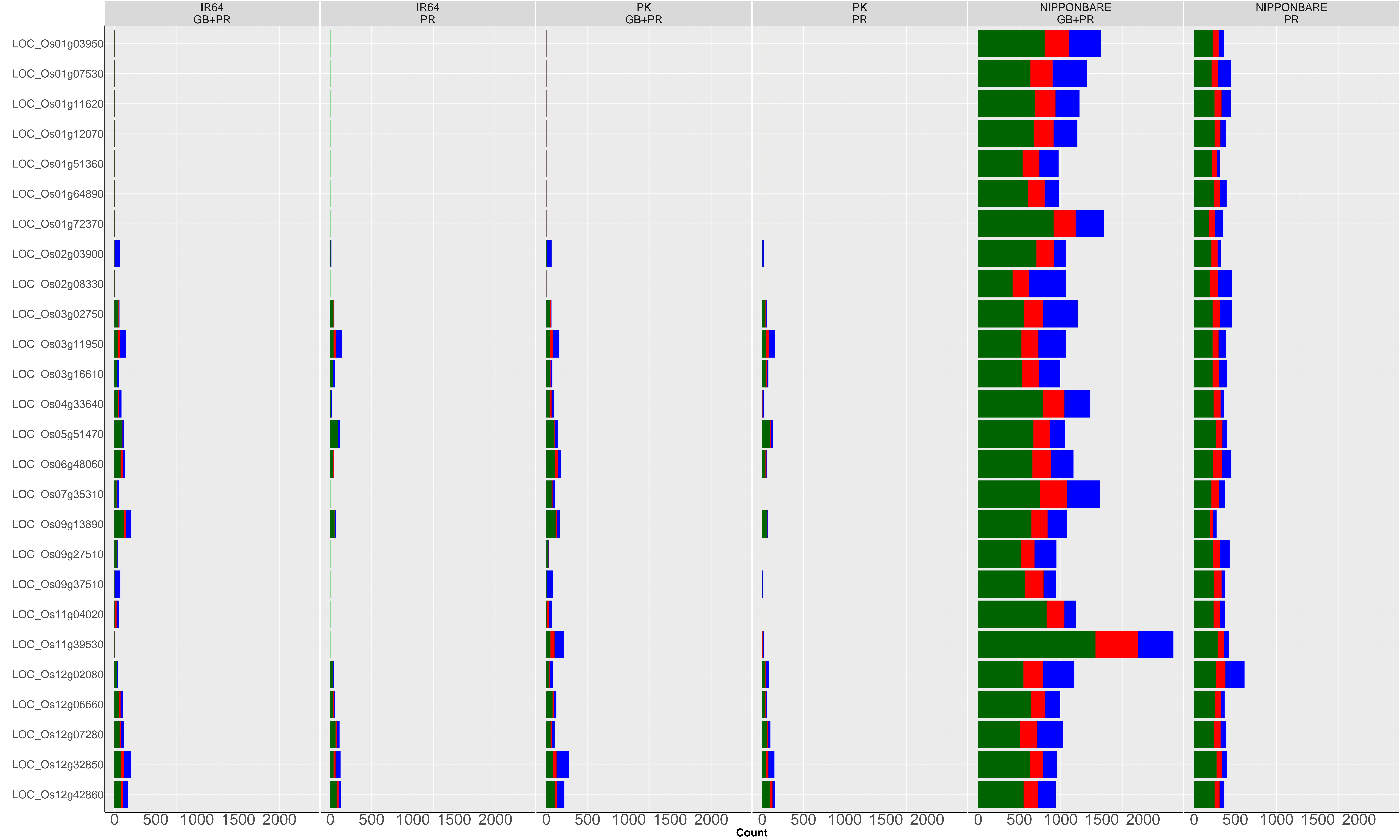
***

***Supplementary figure 1.*** Stacked barplot showing differences among three different cytosine methylated contexts: CG (blue), CHG (red), and CHH (green) per gene in three different rice varieties. The results are discriminated taking into account the location of the epigenetic mark, either in the promoter region (PR) or inside the coding region of analyzed genes.
